# Part-set cuing effects in spatial memory: the role of interitem associations

**DOI:** 10.3389/fpsyg.2024.1364382

**Published:** 2024-05-21

**Authors:** Yufei Zhao, Xiao Hou, Yurong Sun, Fengqiang Gao, Lei Han

**Affiliations:** School of Psychology, Shandong Normal University, Jinan, China

**Keywords:** spatial memory, part-set cuing facilitation effect, part-set cuing impairment effect, interitem associations, miniatures

## Abstract

Part-set cuing facilitation and impairment effects are rarely found in spatial memory, which is a challenge to the theories of part-set cuing effects based on lexical stimulus. This study aims to investigate whether there part-set cuing facilitation and impairment effects are present in spatial memory by constructing two types of memory scenes with high and low degrees of interitem associations, achieved by manipulating the presentation of miniatures. This study examined the effects of different part-set cues on free recall, recognition, and reconstruction tasks. The results of two experiments revealed that matrix cues impaired the performance of three recall tasks in memory scenes with a high degree of interitem associations, and scene cues facilitated the reconstruction performance (Experiment 1). Conversely, in memory scenes with a low degree of interitem associations, the impairment effect of matrix cues was not observed in the three recall tasks, but scene cues still facilitated the reconstruction performance (Experiment 2). These findings supported the retrieval strategy disruption hypothesis, the two-mechanism and the multi-mechanism accounts, demonstrating the significance of interitem associations in spatial memory. Furthermore, the results provided direct evidence for the importance of the encoding-retrieval strategy matching principle in spatial memory tasks.

## 1 Introduction

Part-set cuing impairment effect refers to the counterintuitive finding that retrieval cues often impair recall when those retrieval cues consist of part of the set of to-be-remembered information (Slamecka, 1968). Conversely, if retrieval cues facilitate recall, this phenomenon is known as part-set cuing facilitation effect. Over the years, a number of experimental studies have proved that part-set cuing impairment effect and facilitation effect have been consistently observed in episodic as well as semantic memory (Brown, 1968; Roediger et al., 1977; Nickerson, 1984; Sloman et al., 1991; Peynircioğlu and Moro, 1995; Oswald et al., 2006; Liu and Bai, 2017), in field of metamemory (Rhodes and Castel, 2008), with intralist and extralist cues (Watkins, 1975), in veridical and false memory settings (Kimball and Bjork, 2002; Reysen and Nairne, 2002; Bäuml and Kuhbandner, 2003) and in field of daily life (Bovee et al., 2009).

Different hypotheses have been proposed to explain how part-set cues affect recall. Studies of part-set cuing effects usually attribute impairment mechanisms to the blocking, inhibition, or strategy disruption. The blocking hypothesis (Roediger, 1973; Rundus, 1973) and the retrieval inhibition (RI) hypothesis suggest that presenting part-set cues may lead participants to prioritize the covert retrieval of cues, blocking or inhibiting the recall of target items and reducing the activation of target items (Anderson et al., 1994; Bäuml and Aslan, 2004). The retrieval strategy disruption (RSD) hypothesis suggests that participants form an individual retrieval plan or encoding strategy when remembering items, and at the test, part-set cues would disrupt retrieval by forcing a serial recall order that is inconsistent with the initial retrieval plan; conversely, part-set cues would facilitate retrieval if the presentation order of part-set cues is consistent with the encoding strategy (Basden et al., 1977; Basden and Basden, 1995).

These three hypotheses explain part-set cuing impairment from different perspectives and under different experimental conditions (Bäuml and Aslan, 2006; Lehmer and Bäuml, 2018a; Wallner and Bäuml, 2020), but essential differences exist between them. The two-mechanism account suggests that inhibition is only valid in low associative encoding conditions, referring to participants’ difficulty in establishing a high degree of interitem associations between the items (Bäuml and Aslan, 2006). Thus, the presentation of part-set cues enhances the interitem interference and triggers an impairment mechanism. The Strategy Disruption holds only in high associative encoding conditions, which refers to the condition where participants can quickly establish a high degree of interitem associations between the items. The high degree of interitem associations may form a priority output order, suggesting that disrupting the presentation order of part-set cues triggers strategy disruption (Aslan and Bäuml, 2007). The two-mechanism account concerns the conditions under which the inhibition and strategy disruption assumptions apply.

After the two-mechanism account, the multi-mechanism account (Lehmer and Bäuml, 2018b) also supports the view that different interference mechanisms can be triggered under different encoding conditions, and on the basis of previous studies (Goernert and Larson, 1994; Bäuml and Samenieh, 2012; Bäuml and Schlichting, 2014) put forward a new perspective. This account assumes that part-set cues trigger both impairment mechanisms (inhibition, blocking, and strategy disruption) and facilitation mechanisms (context reactivation), with inhibition and blocking occurring with low associative encoding and strategy disruption with high associative encoding. Impairment mechanisms assert that retrieval cues impair recall when access to the study context at the test is maintained because the impairment mechanism’s relative contribution is more significant than context reactivation’s contribution. When access to the study context is impaired at the test, the relative contribution of context reactivation increases, and part-set cues no longer impair recall. The multi-mechanism account identifies the conditions under which part-set cuing impairment can be transformed into facilitation and provides a more inclusive theoretical framework for research in the field of memory. Currently, more and more studies have supported this account and the new mechanism of context reactivation (John and Aslan, 2018; Kelley and Parihar, 2018; Aslan and John, 2019).

However, different from the context reactivation mechanism, there is another overlooked facilitation mechanism (Basden, 1973; Peynircioğlu, 1989; Sloman et al., 1991; Serra and Nairne, 2000; Basden et al., 2002; Kelley and Bovee, 2007). For instance, Serra and Nairne (2000) placed retrieval cues back into their original serial position or randomly placed them, then marked the original serial position of retrieval cues with “+” signs in the uncued condition. The results showed that the recall of target items was better when retrieval cues were consistent with the original encoding strategy. Other researchers (Basden et al., 2002) also found that when odd or even items at the original positions are provided as cued items, the interitem associations between cued items and target items facilitates recall. This suggests that a successful sequential memory model needs to consider the factor of interitem associations and the way of presentation of the retrieval cues (Kelley and Bovee, 2007).

Similar memory models that take into account both the presentation of cues and interitem associations have also emerged in the study of spatial memory. Some researchers have attempted to investigate the effect of part-set cues on spatial memory using interconnected circuit pieces or chess pieces, and have found that part-set cuing facilitation effect. For example, Cole et al. (2013) conducted a study in which participants were asked to reconstruct an inter-connected Snap Circuit object. It was found that the cued bottom-layer pieces facilitated memory of the spatial positions of both the bottom- and top-layer pieces in a Snap Circuit object and that the position-only cues without piece identity were still useful compared to the condition in the absence of cues. Kelley et al. (2016) subsequently examined the effects of connection type (connected vs. unconnected) and cue type (cued vs. uncued) on spatial memory and found that part-set cuing facilitation effect existed only when the stimuli were connected. The essence of the interconnected feature of the stimulus is to enhance the degree of interitem associations. From these two studies, we can speculate that the degree of interitem associations is an important factor affecting the existence of part-set cuing effects in spatial memory.

However, it should be noted that only a few studies on spatial memory have found part-set cuing facilitation effect, but not part-set cuing impairment effect. Some researchers have used different stimulus materials to verify the part-set cuing impairment effect, and found that part-set cues did not impair recall (Watkins et al., 1984; Huffman et al., 2001; Drinkwater et al., 2006; Fritz and Morris, 2015). For example, Watkins et al. (1984) conducted a study in which ten chess games with 24 chess pieces were randomly divided into two sets of 12, with one of the sets randomly chosen as the cue set. However, the study found that part-set cues did not facilitate or impair recall of the remaining pieces. Similarly, Huffman et al. (2001) and Drinkwater et al. (2006) conducted separate studies examining the influence of part-set cuing on chess positions using chunks in the cue set and moderating cue-set size, respectively. The results of both studies were consistent with the Watkins et al.’s study, which showed no effect of part-set cuing. It may be mentioned that the part-set cuing impairment effect has not been found in spatial memory studies, which is a curious phenomenon that is difficult to explain. And this result is a challenge to the theories of part-set cuing effects based on lexical stimulus. Some researchers have shown that this is related to the semantic information contained in chess stimuli (e.g., rook, knight) (Cole et al., 2013) and/or the degree of interitem associations (Kelley et al., 2016). In summary, if we want to continue investigating the effect of part-set cues on spatial memory, we must focus on influential factors such as the identity (semantic information), the presentation of retrieval cues and the mechanism of interitem associations.

The degree of interitem associations formed during encoding is a factor crucial in determining whether retrieval strategy disruption or retrieval inhibition would influence performance (Bäuml and Aslan, 2006). In previous studies on spatial memory, researchers chose different experimental materials or set different cuing conditions, and the factor of interitem associations was not given enough attention, so the results were inconsistent. If we continue to select chess pieces as learning material, we have to consider the move rules of chess pieces and the possible empirical knowledge of the participants. In addition, previous studies often used only a single memory task to examine the effect of part-set cuing on spatial memory, such as recalling only the name of the target item and ignoring the possible role of the position. For example, Fritz and Morris (2015) set room scene and matrix scene with different degree of interitem associations to investigate the recall of the name of the target objects after presenting the cued objects. In the room scene, one item had salient spatial relationships with several other items (e.g., chair next to table and wallet), and the degree of interitem associations was high. In the matrix scene, the individual objects were placed in a two-dimensional matrix, and the degree of interitem associations was low. The results showed that more names of objects were recalled from the room scene than from the matrix scene, indicating that the scene’s organization aided memory, but the cues did not assist recall. And the results did not change after increasing the learning difficulty of the room scene. As the author says, this lack of effect may be related to the spatial nature of the task. We believe that in the spatial memory tasks, besides recalling the names of the items, it is also necessary to investigate the recognition of the items and the reconstruction of their positions. In other words, these three different recall tasks involving free recall, recognition, and reconstruction should be set at the test phase. To a certain extent, the setting of the three kinds of recall tasks allows to separate the identity information contained in the items from the spatial position information, thus achieving the goal of examining separately the effect of part-set cues on the identity or position of the target items. In the free recall task, participants first recall the names of the target items. This task considers that participants does not ignore the identity of the item itself while memorizing the scene. The free recall task is followed by the recognition task, which mainly assesses the familiarity of the items. The reconstruction task is to place the target items in their original positions, which mainly considers that the participants also process the positions of the items while memorizing the scene.

Based on the above discussion, this study selects miniatures as experimental materials to construct memory scenes with a high degree of interitem associations (Experiment 1) and memory scenes with a low degree of interitem associations (Experiment 2) respectively, and examines the effects of different cuing conditions on spatial memory through three recall tasks (free recall, recognition, and reconstruction). On the one hand, the reconstruction task of memory scenes is not affected by the rules, such as the move rules of chess pieces. On the other hand, memory scenes constructed from miniatures are closer to real-life spatial memory and have elevated ecological validity.

## 2 Experiment 1

In Experiment 1, matrix cues were inconsistent with the encoding strategy, and scene cues were consistent with the encoding strategy. This experiment constructed memory scenes with a high degree of interitem associations, aimed to examine whether a part-set cuing impairment effect would arise in the matrix condition and whether a part-set cuing facilitation effect would occur in the scene condition.

### 2.1 Materials and methods

#### 2.1.1 Participants

A sample size of *n* = 19 is sufficient to detect a medium effect size of *f* = 0.27 [the effect of no part-list cuing vs. standard part-list cuing vs. self-paced part-list cuing conditions from Wallner and Bäuml (2021)], with *α* = 0.05 and 1 − β = 0.95. In total, 35 Chinese college students (8 males, 27 females) with an average age of 20.89 ± 1.30 years were recruited for Experiment 1. All participants had normal or corrected visual acuity, realized no color blindness, and had not participated in a similar memory experiment. This study was conducted with the approval of the local ethics committee. Participants signed an informed consent form prior to participating in this experiment and received a reward upon completion.

#### 2.1.2 Materials

Miniatures were chosen for the stimulus materials, and other materials included a sky-blue sandbox (60 cm × 75 cm), green quartz sand, sand tools, and one set of supporting tools (including a sand shovel and sand scraping tool).

Three scene types (zoo scene, palace scene, war scene) were identified first, based on the types of available miniatures (animal miniatures, palace doll miniatures, and weapon miniatures have the most significant number of miniatures and ample selection space for miniatures). The miniatures with unique materials (such as springs), multiple information (such as lovers’ dolls), and difficult-to-name miniatures were excluded. Ultimately, 16 representative miniatures were retained for each scene type, including eight target miniatures and eight cued miniatures. Next, three college students who did not participate in the formal experiment were selected to complete the placement of three scene types, for a total of nine scenes. The placement of memory scenes required to have a high degree of associations between the miniatures. Besides placing the 16 representative miniatures, the students could also move sand or add grass and trees to enhance the meaningfulness of the scenes. Additionally, another 24 college students were required to rate the degree of interitem associations between the miniatures (with 1 being the lowest and 5 being the highest). After the ratings, only one memory scene with a degree of associations between miniatures ≥4.0 was kept in each scene type (zoo scene, palace scene, war scene). There was no significant difference between three memory scenes, *F*s < 1, *p*s > 0.05.

#### 2.1.3 Design

A 3 (cuing condition: background condition, matrix condition, scene condition) × 3 (recall task: free recall, recognition, reconstruction) within-subjects experimental design was adopted.

**Figure 1:**
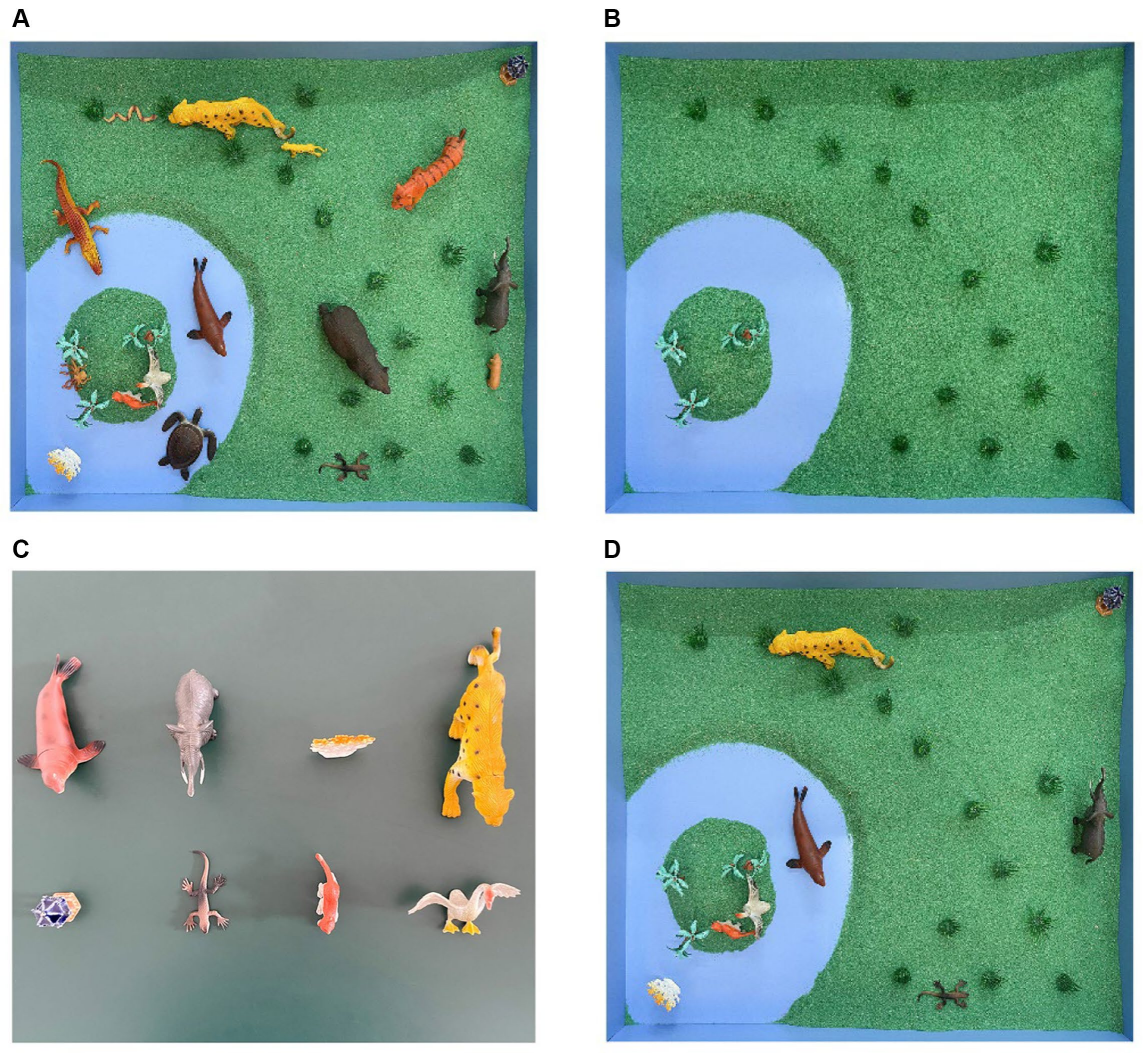
Take the zoo scene **(A)** of the 3 scene types as an example, the background **(B)**, matrix cues **(C)**, and scene cues **(D)** in Experiment 1.

The cuing condition contained three levels. As shown in Figure 1, taking the zoo scene as an example (see Figure 1A), the specific differences between the three cuing conditions were as follows: (1) The background condition: No retrieval cues were given in the free recall and recognition tasks, and the background was presented in the reconstruction task. The background referred to the remaining scenes after 16 miniatures were removed from the memory scene (see Figure 1B). (2) The matrix condition: In three recall tasks, the cued miniatures were presented in a 2 × 4 matrix on a desktop outside the sandbox (see Figure 1C), and the background was also provided in the reconstruction task. (3) The scene condition: Scene cues were presented in three recall tasks. Scene cues referred to the remaining scenes after the target miniatures were taken from the memory scene, including the background and the cued miniatures placed in their original position (see Figure 1D). Compared to matrix cues, scene cues matched the encoding strategy formed by participants when learning the memory scenes.

The dependent variables in the study were the participants’ free recall performance of the target miniatures in each scene (the correct number of target miniatures recalled divided by the total number of target miniatures to be recalled), the recognition performance of the target miniatures (the correct number of target miniatures recognized divided by the total number of target miniatures to be recognized), and the reconstruction recall performance of the target miniatures (the number of correctly placed target miniatures divided by the total number of target miniatures to be placed). The calculation method of the target variable was consistent with previous research (Slamecka, 1968). In order to control for individual differences, the sequential effect of memory scenes, and the correspondence between memory scenes and cuing conditions, a Latin square balance was performed between three memory scenes and three cue conditions.

#### 2.1.4 Procedure

The formal experiment consisted of three blocks. Participants only needed to complete one block containing three cuing conditions. There were three recall tasks for each cuing condition, and the order of the three recall tasks was free recall, recognition, and reconstruction. Before the experiment, participants were informed that they did not need to remember the two types of miniatures, grass and trees, and used their own methods to remember all the miniatures. First, a scene (e.g., zoo scene) was presented for 1 min. Second, participants completed a numerical computation task on the paper for 1 min. Finally, participants were asked to perform free recall, recognition, and reconstruction tasks sequentially. In the free recall, participants were presented with one cuing condition (e.g., background condition) and then asked to write the names of the miniatures; in the recognition task, participants were shown all the miniatures they had learned and those they had not, and were asked to choose which one they had just learned; in the reconstruction task, the miniatures from the recognition task were used to reconstruct the newly learned memory scene. To avoid fatigue effects on the participants, they took a 3-min break after completing all recall tasks in each memory scene.

#### 2.1.5 Data analysis

G*Power 3.1.9.6 software (Faul et al., 2009) was used to calculate the minimum sample size, and SPSS 21 statistical package (IBM, Armonk, USA) was employed to examine the datasets.

### 2.2 Results

The correct recall performance of the participants in the three cuing conditions under the free recall, recognition, and construction tasks was shown in Table 1.

**Table 1:** Means and standard deviations of target items recalled by cuing condition and recall task.

	Free recall	Recognition	Reconstruction
Background condition	0.75 ± 0.13	0.81 ± 0.10	0.66 ± 0.17
Matrix condition	0.62 ± 0.22	0.74 ± 0.16	0.56 ± 0.23
Scene condition	0.78 ± 0.13	0.84 ± 0.13	0.78 ± 0.17

**Figure 2:**
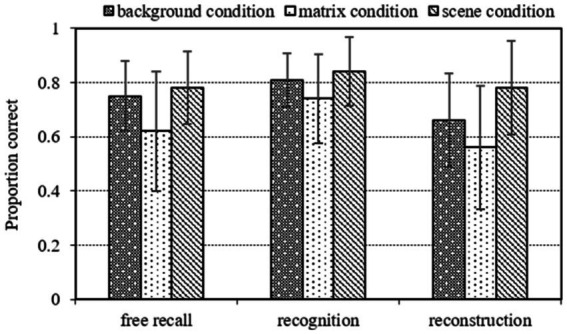
Mean proportion correct recall for the target items as function of cuing condition (background condition, matrix condition, scene condition) and recall task (free recall, recognition, reconstruction) in Experiment 1. The error bars indicate standard error.

Figure 2 displays the mean proportion correct recall as a function of cuing condition and recall task. A repeated-measures analysis of variance (ANOVA) revealed that the main effect of cuing condition was significant [*F*(2, 68) = 15.73, *p* < 0.001, $\eta_{p}^{2}$ = 0.32], the recall performance of matrix condition was significantly lower than that of background condition (*p* < 0.001) and scene condition (*p* < 0.001), and there was no significant difference in recall performance between background condition and scene condition (*p* = 0.051). The main effect of recall task was significant [*F*(2, 68) = 47.75, *p* < 0.001, $\eta_{p}^{2}$ = 0.58]. The interaction between cuing condition and recall task was significant [*F*(4, 136) = 4.77, *p* = 0.001, $\eta_{p}^{2}$ = 0.12]. Further analysis revealed that in the free recall task, the free recall performance of matrix condition was significantly lower than that of background condition [*t*(34) = 3.66, *p* < 0.001, Cohen’s *d* = 0.62] and scene condition [*t*(34) = 4.24, *p* < 0.001, Cohen’s *d* = 0.72], and there was no significant difference in free recall performance between background condition and scene condition [*t*(34) = 0.82, *p* = 0.419, Cohen’s *d* = 0.14]. In the recognition task, the recognition performance of matrix condition was significantly lower than that of background condition [*t*(34) = 2.65, *p* = 0.012, Cohen’s *d* = 0.45] and scene condition [*t*(34) = 3.41, *p* = 0.002, Cohen’s *d* = 0.58], and there was no significant difference in recognition performance between background condition and scene condition [*t*(34) = 1.07, *p* = 0.294, Cohen’s *d* = 0.18]. In the reconstruction task, the reconstruction performance of matrix condition was significantly lower than that of background condition [*t*(34) = 2.67, *p* = 0.012, Cohen’s *d* = 0.45] and scene condition [*t*(34) = 5.82, *p* < 0.001, Cohen’s *d* = 0.98], the reconstruction performance of scene condition was significantly higher than that of background condition [*t*(34) = 2.98, *p* = 0.005, Cohen’s *d* = 0.50].

### 2.3 Discussion

Experiment 1 constructed three memory scenes with a high degree of interitem associations, and three recall tasks of free recall, recognition and reconstruction were used to investigate the influence of different cuing conditions (background condition, matrix condition, scene condition) on spatial memory. The results reflected that the matrix cues disrupted the original organized encoding strategy, resulting in poor recall performance. Moreover, the scene cues were consistent with the original organized encoding strategy, which would help activate the encoding strategy and improve recall performance. These results supported the RSD hypothesis (Basden et al., 1977; Basden and Basden, 1995; Bäuml and Aslan, 2006). From the above results, when learning and remembering a spatial memory scene, the participants may encode not only the identity (semantic information) of miniatures, but also the position information of miniatures (spatial processing) and the associations between miniatures in spatial tasks, so that participants could benefit from scene cues. In the testing phase, the free recall task was only able to investigate the retrieval of the miniature’s identity, while it was difficult to comprehensively investigate the retrieval of the miniature’s spatial position. Therefore, in addition to the classical free recall task, it was highly desirable to set up recognition and reconstruction tasks to examine the familiarity of participants with the miniatures and the retrieval of their spatial positions. Experiment 1 showed that there were part-set cuing impairment effect and part-set cuing facilitation effect in spatial memory, which provided evidence for the existence of part-set cuing impairment in spatial memory.

## 3 Experiment 2

Conventional accounts of memory assume that associations among memorized items will cue recall (Raaijmakers and Shiffrin, 1981; Anderson, 1983; Ebbinghaus, 2013). When the degree of interitem associations among the items is low, as participants should experience high levels of interitem interference (Bäuml and Aslan, 2006). Experiment 1 constructed three scenes with a high degree of interitem associations and first verified the existence of part-set cuing impairment and facilitation effects in spatial memory. Based on the results of Experiment 1, Experiment 2 constructed three scenes with a low degree of interitem associations, and continued to use three recall tasks to test whether there were stable part-set cuing impairment and facilitation effects.

### 3.1 Materials and methods

#### 3.1.1 Participants

A sample size of *n* = 19 is sufficient to detect a medium effect size of *f* = 0.27 [the effect of no part-list cuing vs. standard part-list cuing vs. self-paced part-list cuing conditions from Wallner and Bäuml (2021)], with *α* = 0.05 and 1 − β = 0.95. Experiment 2 recruited 35 Chinese college students (11 males, 24 females) with an average age of 19.91 ± 1.74 years. All participants had normal or corrected visual acuity, realized no color blindness, and had not participated in a similar memory experiment. This study was conducted with the approval of the local ethics committee. Participants signed an informed consent form prior to participating in this experiment and received a reward upon completion.

#### 3.1.2 Materials

The backgrounds in three memory scenes (Experiment 1) with a high degree of interitem associations were used as the backgrounds in three memory scenes (Experiment 2) with a low degree of interitem associations to avoid the possible influence of different backgrounds. In three memory scenes with a low degree of interitem associations, 16 representative miniatures were presented in a 4 × 4 matrix (Fritz and Morris, 2015). Then, only one scene with a degree of interitem associations ≤3.5 remained in each scene type (zoo scene, palace scene, and war scene). There was no significant difference between three memory scenes with a low degree of interitem associations, *F*s < 1, *p*s > 0.05. Moreover, there was a significant difference in memory scenes with different degrees of interitem associations [*F*(1, 46) = 11.44, *p* = 0.001].

#### 3.1.3 Design and procedure

**Figure 3:**
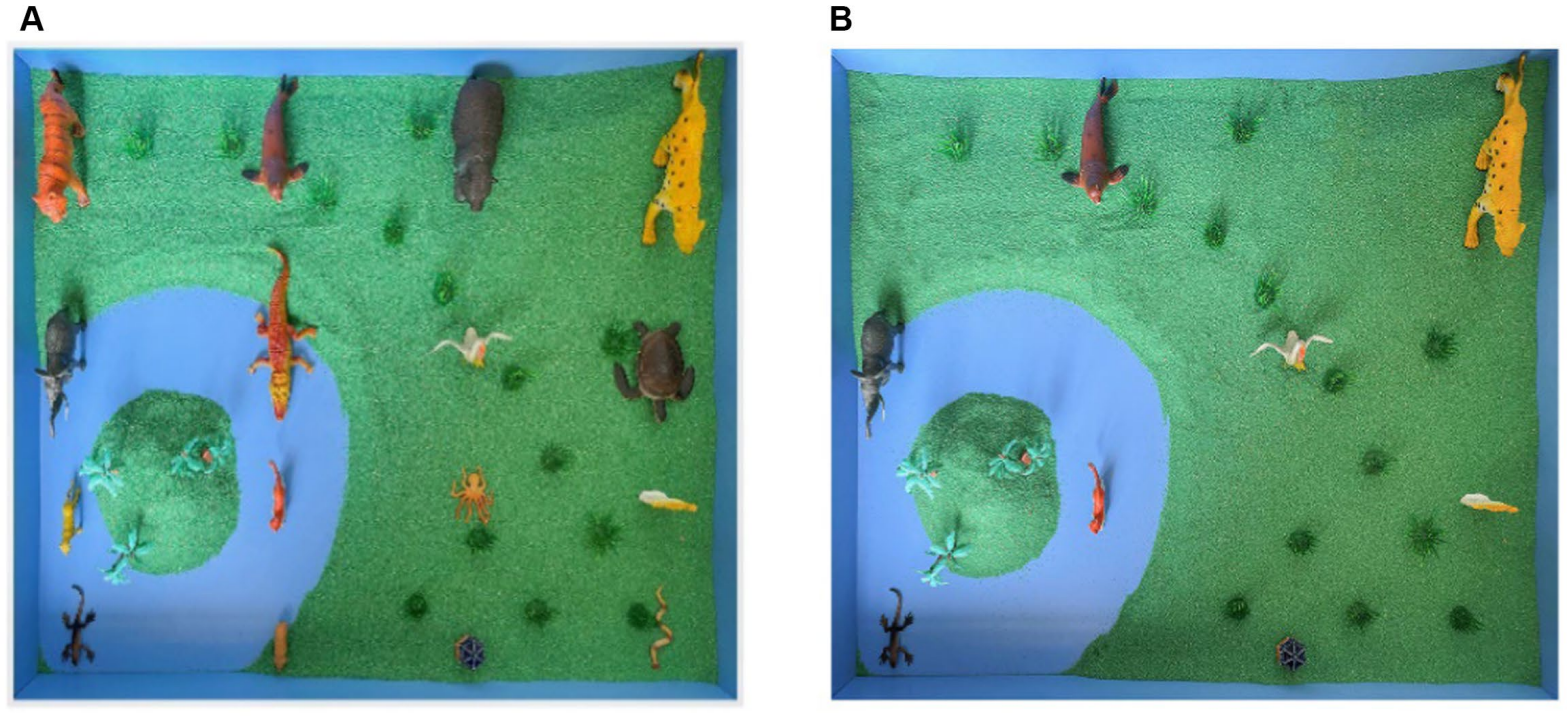
Sixteen representative animal miniatures with 4 × 4 matrix arrangement to form the zoo scene **(A)** with a low degree of interitem associations, and the scene cues refer to the remaining scenes after removing the target animal miniatures **(B)**.

A 3 (cuing condition: background condition, matrix condition, scene condition) × 3 (recall task: free recall, recognition, reconstruction) within-subjects experimental design was adopted. As shown in Figure 3, taking the zoo scene with a low degree of interitem associations as an example (see Figure 3A), the details of the cuing conditions were set the same as in Experiment 1 (see Figure 3B for scene cues). The data statistics of the dependent variables and the experimental procedure were the same as Experiment 1.

#### 3.1.4 Data analysis

G*Power 3.1.9.6 software (Faul et al., 2009) was used to calculate the minimum sample size, and SPSS 21 statistical package (IBM, Armonk, USA) was employed to examine the datasets.

### 3.2 Results

The correct recall performance of the participants in the three cuing conditions under the free recall, recognition, and construction tasks was shown in Table 2.

**Table 2:** Means and standard deviations of target items recalled by cuing condition and recall task.

	Free recall	Recognition	Reconstruction
Background condition	0.71 ± 0.15	0.80 ± 0.15	0.61 ± 0.20
Matrix condition	0.69 ± 0.17	0.81 ± 0.16	0.60 ± 0.21
Scene condition	0.68 ± 0.15	0.81 ± 0.15	0.73 ± 0.22

**Figure 4:**
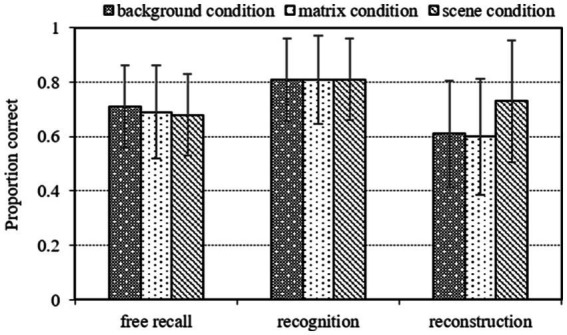
Mean proportion correct recall for the target items as function of cuing condition (background condition, matrix condition, scene condition) and recall task (free recall, recognition, reconstruction) in Experiment 2. The error bars indicate standard error.

Figure 4 displays the mean proportion correct recall as a function of cuing condition and recall task. A repeated-measures analysis of variance (ANOVA) revealed that the main effect of cuing condition was not significant [*F*(2, 68) = 1.02, *p* = 0.367]. The main effect of recall task was significant [*F*(2, 68) = 82.32, *p* < 0.001, $\eta_{p}^{2}$ = 0.71]. The interaction between cuing condition and recall task was significant [*F*(4, 136) = 5.97, *p* < 0.001, $\eta_{p}^{2}$ = 0.15]. Further analysis revealed that in the free recall task, there was no significant difference in free recall performance between the three cuing conditions (*p*s > 0.05). In the recognition task, there was no significant difference in recognition performance between the three cuing conditions (*p*s > 0.05). In the reconstruction task, the reconstruction performance of scene condition was significantly higher than that of background condition [*t*(34) = 2.41, *p* = 0.021, Cohen’s *d* = 0.41] and matrix condition [*t*(34) = 2.41, *p* = 0.022, Cohen’s *d* = 0.41], and there was no significant difference in reconstruction performance between background condition and matrix condition [*t*(34) = 0.19, *p* = 0.854, Cohen’s *d* = 0.03].

### 3.3 Discussion

Experiment 2 found that part-set cuing impairment effect (Experiment 1) that existed in the free recall task and recognition task in memory scenes with a high degree of interitem associations disappeared in memory scenes with a low degree of interitem associations. However, in the reconstruction task, scene cues still facilitated the reconstruction performance, which reflected part-set cuing facilitation effect. The finding of part-set cuing facilitation effect was consistent with previous studies (Cole et al., 2013). We suggested that the disappearance of the part-set cuing impairment effect may be related to the increased difficulty of the task. In scenes with a low degree of interitem associations, participants may sacrifice encoding the identity of miniatures and focus more on their spatial positions. However, the free recall task examined the retrieval of the identity of miniatures. The encoding strategy was inconsistent with the retrieval strategy, which increased the retrieval difficulty and thus the part-set cuing impairment effect disappeared. In the reconstruction task, where scene cues were consistent with the original organized encoding strategy and the participants could reset the target miniatures based on the relationship between the front, back, left and right sides of the miniatures, scene cues were likely to exert maximum positive values and therefore facilitate recall.

## 4 General discussion

This study used miniatures to construct memory scenes and explored the effect of different cuing conditions on spatial memory using three recall tasks. Experiment 1 first constructed three memory scenes with high degree of interitem associations to test whether there were part-set cuing impairment and facilitation effects in spatial memory. As a result, it was found that matrix cues (a 2 × 4 matrix) played an impairment role in three recall tasks, while scene cues (background, cued miniatures and their positions) played a facilitation role in the reconstruction task. These findings suggest that when scene cues are consistent with the retrieval strategy, the likelihood of scene cues becoming effective cues is high, and it is easy to induce part-set cuing facilitation effect. When matrix cues are inconsistent with the retrieval strategy, the probability of matrix cues becoming effective cues is low, and it is more likely to induce part-set cuing impairment effect. These results are consistent with some of the conclusions of previous studies (Cole et al., 2013; Kelley et al., 2016), and support RSD hypothesis, the two-, and the multi-mechanism hypothesis (Basden et al., 1977; Basden and Basden, 1995; Bäuml and Aslan, 2006; Lehmer and Bäuml, 2018a,b). When learning a memory scene with a high degree of interitem associations, participants may not only memorize the identity and position of miniatures but also form their own organized memory encoding strategy based on the associations between the miniatures. In memory scenes with a high degree of interitem associations, there is a high degree of associations between miniatures, which minimizes the negative influence of the RI mechanism so that performance is driven by the RSD mechanism. That is, the retrieval performance is best when the scene cues are consistent with the encoding strategy. And the matrix cues are inconsistent with the encoding strategy, the retrieval performance is worst.

The early Encoding Specificity Principle is consistent with the Strategy Disruption hypothesis. According to the Encoding Specificity Principle (Tulving and Thomson, 1973), recall performance is best when the context at encoding matches that at retrieval. The reconstruction task involves participants placing target miniatures in their original positions after recognition, so the reconstruction process is highly similar to the encoding process. The scene cues in the reconstruction task contain background information, cued miniatures and their position information, which can maximize the positive value of the retrieval cues. When providing scene cues (background, cued miniatures and their positions) in the reconstruction task, scene cues are sufficiently special and clear. At this time, the participants can fully utilize the association characteristics between target items and cued items formed in the encoding phase to exclude other possible alternative items and incorrect positions in the retrieval phase and successfully retrieve. This research has demonstrated the importance of the presentation way of retrieval cues. Complete retrieval cues, such as the background, cued miniatures and their correct positions processed together in the encoding phase, are provided in an ordered manner in the retrieval phase, which would improve the matching between encoding and retrieval, then thus facilitate recall.

Experiment 2 continued to examine the stability of part-set cuing impairment and facilitation effects in memory scenes with a low degree of interitem associations. It was found that part-set cuing impairment effect disappeared in the free recall and recognition tasks, while part-set cuing facilitation effect remained stable in the reconstruction task. In memory scenes with a low degree of interitem associations, there is a low degree of associations between miniatures, which can induce the RI mechanism. However, scene cues are consistent with the encoding strategy, which enable the original organized encoding strategy to be largely restored. In this case, the positive effect of the RSD is higher than the damaging effect of the RI, resulting in a part-set cuing facilitation effect. However, there was no part-set cuing impairment effect in memory scenes with a low degree of interitem associations. This may be because in scenes with a low degree of interitem associations, participants find it difficult to effectively integrate information such as background and the identity and the position of miniatures to complete a deep processing of memory scenes. In order to optimize the encoding and retrieval of memory scenes in a limited amount of time, participants may sacrifice their processing of the miniature’s identity and instead focus on the miniatures’ spatial position, thus forming spatial encoding strategies for the relationship between miniatures, front, back, left, and right. When the encoding strategy is inconsistent with the retrieval strategy, it increases the retrieval difficulty, and thus the part-set cuing impairment effect vanishes. Moreover, due to the unique nature of the background, participants may not only encode miniatures when memorizing the scene, but also the background. In Experiment 1, the background serves as a helpful cue to enhance the degree of interitem associations between the miniatures. But the background may transform into an unfavorable cue in Experiment 2. Further research is needed to explain the role of background.

The result of part-set cuing facilitation effect in spatial memory was congruent with the part-set cuing facilitation demonstrated in serial order memory (Sloman et al., 1991; Serra and Nairne, 2000; Basden et al., 2002; Kelley and Bovee, 2007). This research has demonstrated the importance of the degree of interitem associations between the items in spatial memory. In spatial memory, in order to examine part-set cuing impairment and facilitation effects, it is necessary to improve the degree of interitem associations between the items so that participants can deeply process the learned materials and form organized encoding strategies during encoding. At the same time, attention should also be paid to the degree of matching between the encoding and retrieval phases. On the one hand, we need to consider whether the presentation of the retrieval cues is consistent with the encoding phase. On the other hand, we should focus on the matching of encoding and retrieval. In other words, in a spatial memory scene where participants will automatically encode the identity of miniatures and their spatial position information, then using only one memory task does not fully evaluate the retrieval performance of participants.

For example, no part-set cuing effect has been found in spatial memory studies using chess pieces as stimulus materials, possibly because the chess has an easily named nature (e.g., king, queen, pawn, horse) and therefore encourages more verbal strategies. On the other hand, the chessboard reconstruction task is to retrieval the spatial position of the chess, which relies on the spatial strategy and ignores the interference effect that the semantic information of the item may play (Cole et al., 2013; Kelley and Parihar, 2018). In addition, Fritz and Morris (2015) studied the part-set cuing effect of objects presented in scene condition or matrix condition and found that there was almost no cuing effect. Although some studies have speculated that this result may be related to the inconsistency of semantic encoding strategy and spatial retrieval strategy (Cole et al., 2013; Kelley et al., 2016), but there is no direct evidence. This study is the first to use miniatures to construct spatial memory scenes with varying degrees of interitem associations, using three recall tasks to comprehensively investigate the role of semantic information and spatial location information of part-set cues on retrieval. The results showed that there are part-set cuing impairment and facilitation effect in spatial memory. The findings provide direct evidence for the importance of the encoding-retrieval strategy matching principle in spatial memory tasks, suggesting that the setting of memory tasks is crucial in spatial memory, and proving that the degree of interitem association is also an influential factor affecting the existence of part-set cuing effects in spatial memory. To be more precise, when examining the impairment and facilitation effect of part-set cuing in spatial memory, both free recall task and recognition task can be used to investigate participants’ retrieval of the identity and familiarity of miniatures, and then the reconstruction task can be used to continue to investigate participants’ retrieval of the position information of miniatures.

There are some limitations to this study. First, it is not easy to guarantee which memory strategies are used by the participants. Further research could consider the use of unified memory strategies, such as using miniatures to create stories. In addition, the same background used in memory scenes with different degrees of interitem associations may overlook the role of the background. It is unclear whether the background plays a positive or negative role in memory scenes with a low degree of interitem associations, and whether it increases or decreases the difficulty of remembering a memory scene. Future research can examine the effect of different cuing conditions on free recall, recognition, and reconstruction tasks in the absence of background.

## Data availability statement

The datasets presented in this study can be found in online repositories. The names of the repository/repositories and accession number(s) can be found at: https://osf.io/sa27p/.

## Ethics statement

The studies involving humans were approved by School of Psychology, Shandong Normal University. The studies were conducted in accordance with the local legislation and institutional requirements. The participants provided their written informed consent to participate in this study.

## Author contributions

YZ: Writing – review & editing, Writing – original draft, Visualization, Validation, Methodology, Investigation, Formal analysis, Data curation. XH: Writing – original draft, Validation, Methodology, Investigation, Formal analysis, Data curation. YS: Writing – original draft, Validation, Investigation, Formal analysis, Data curation. FG: Writing – review & editing, Supervision, Resources, Project administration, Methodology, Funding acquisition, Conceptualization. LH: Writing – review & editing, Supervision, Resources, Project administration, Methodology, Funding acquisition, Conceptualization.
